# Lysophosphatidic acid promotes lipid metabolism through AMPK signaling to support Coxsackievirus infection

**DOI:** 10.1128/jvi.00395-26

**Published:** 2026-07-16

**Authors:** Natalie J. LoMascolo, Caroline E. Thomas, Madison L. Lange, Bryan C. Mounce

**Affiliations:** 1 Department of Microbiology and Immunology, Loyola University Chicago Stritch School of Medicine 548051, Maywood, Illinois, USA; 2 Infectious Diseases and Immunology Research Institute, Maywood, Illinois, USA; St Jude Children's Research Hospital, Memphis, Tennessee, USA

**Keywords:** lysophosphatidic acid, lysophosphatidic acid receptor 1, Coxsackievirus B3, AMPK, lipids

## Abstract

Lysophosphatidic acid (LPA) is a bioactive signaling lipid that regulates cellular processes, including growth, survival, differentiation, and metabolic homeostasis. LPA signals through its G-protein-coupled receptor, LPA receptor 1 (LPAR1), to modulate pathways controlling cellular signaling and metabolism. Viruses rely on cellular metabolism for efficient replication, and lipids function at nearly every stage in virus replication. Using Coxsackievirus B3 (CVB3) as a model, we performed global metabolomic analysis of infected Huh7 cells and identified lipid classes altered upon infection. Using small-molecule inhibitors targeting lipids, we identified LPA signaling as a key factor in CVB3 infection. Inhibiting LPA signaling with AM095 or via siRNA knockdown reduces CVB3 infection by over 100-fold. We find that LPA mediates CVB3 genome replication. Mechanistically, LPA signaling through LPAR1 facilitates cellular lipid synthesis by regulating AMPK and downstream enzymes, including fatty acid synthase (FASN) and acetyl-CoA carboxylase 1 (ACC1). In AM095-treated cells, lipid droplets are depleted, and cells exhibit a quiescent-like phenotype. Replenishing lipids fully rescues viral replication, indicating that LPA’s proviral function is to facilitate lipid synthesis through AMPK signaling. These findings identify LPA-LPAR1 signaling as a regulator of host lipid metabolism that supports CVB3 replication, revealing a metabolic axis for antiviral intervention.

**IMPORTANCE:**

Cellular metabolism supports virus infection by providing energy and the building blocks for virus replication. During viral infection, cellular metabolism changes drastically, and we find that lipids are among the most significantly changed metabolites during infection with Coxsackievirus B3, a picornavirus. We used metabolomics to characterize changes in metabolites in infected cells, and we followed up on these data with a drug screen with molecules targeting cellular lipid pathways. We identified lysophosphatidic acid (LPA) signaling as a major regulator of Coxsackievirus infection, supporting viral genome replication via lipid synthesis. This study underscores the importance of lipids in viral infection, uncovers new mechanisms by which LPA signals, and provides new insight into LPA’s function in Coxsackievirus infection.

## INTRODUCTION

Lysophosphatidic acid (LPA) is a bioactive phospholipid that plays a central role in coordinating cellular growth, survival, migration, and metabolic regulation. LPA is generated primarily through the enzymatic activity of autotaxin and signals extracellularly through a family of G-protein-coupled receptors (LPAR1–LPAR6) (1). Engagement of these receptors activates diverse downstream pathways, including Rho/ROCK, PI3K–AKT, and MAPK signaling, enabling LPA to influence cytoskeletal dynamics, transcriptional programs, and metabolic flux. Through these signaling networks, LPA regulates developmental processes, tissue repair responses, and immune cell behavior, while dysregulated LPA signaling has been implicated in cancer progression, fibrosis, neuropathic pain, and inflammatory disease. Thus, LPA serves as a critical lipid mediator linking extracellular cues to intracellular programs that control cell fate and functional state.

Previous studies have observed anti- and proviral capacities of LPA (2, 3), including a role for LPA in the immune response to viral infection (1). Although the scope of LPA signaling is not fully defined, previous studies have linked increased LPA levels to hepatitis C virus (HCV) and SARS-CoV-2 infection. In these cases, viral infection led to increased expression of LPA receptors (LPARs), which promoted immune responses by activating interferon signaling pathways. Specifically, LPAR signaling was shown to induce the translocation of IRF3, a transcription factor involved in interferon signaling (2). However, it is unknown whether a similar mechanism applies to other viruses or if LPA signaling influences viral clearance through other pathways, such as shifts in metabolism. The spectrum of mechanisms by which LPA influences infection, particularly its dual roles as both an antiviral and proviral factor, remain unknown.

Cells undergo extensive metabolic reprogramming during virus infection, which has been observed across various types of viruses, including enteroviruses (4, 5). Viral reprogramming of cellular metabolism involves the ability to manipulate host cell machinery, hijack essential structural components, and reshape the overall metabolite profile of the cell (6). One virus that is particularly effective at this is Coxsackievirus B3 (CVB3). CVB3 is a positive-sense, single-stranded RNA virus that poses a significant threat to human health (7). As a member of the enterovirus genus within the *Picornaviridae* family, CVB3 is known to cause severe disease such as myocarditis, which in some cases progresses to heart failure, where transplantation becomes the only viable treatment (7). Despite the severity of CVB3-associated disease, no approved antiviral treatments or vaccines are currently available.

CVB3 reprograms host metabolism to support its replication; thus, targeting metabolic pathways offers a potential new strategy for antiviral development. Previous work has consistently illuminated a critical role for lipid metabolism in CVB3 infection, including viral entry, replication complex formation, and energy manipulation (4, 8, 9). For example, CVB3 is known to utilize lipids, particularly cholesterol, during cellular attachment (10). RNA viruses replicate on specialized membrane structures derived from host organelles such as the ER or mitochondria (11, 12). These replication organelles (ROs) form invaginated vesicles or double-membrane vesicles (DMVs) (13), creating pouch-like or separated structures, respectively, that provide a protected environment for RNA replication. CVB3 replicates within DMVs (13). To form these ROs, viruses hijack host lipid synthesis machinery. Lipids, such as cholesterol, must be generated to maintain the proper membrane composition and fluidity. Specifically, CVB3 enhances lipid synthesis by upregulating the MEK/ERK signaling pathway, which increases production of the transcription factor SREBP1a (14). Studies have shown that the CVB3 viral protease 2A enhances SREBP1a promoter activity (14). Once lipid synthesis is upregulated, these newly generated lipids must be recruited to the ROs. CVB3 recruits cholesterol by using its viral protein 2BC, which activates clathrin-mediated endocytosis (CME) to shuttle cholesterol from the plasma membrane into the cytoplasm (15–17). CVB3 then recruits phosphatidylinositol 4-kinase III beta (PI4KIIIB), which binds directly to Rab11 and delivers it to the sites of replication (15). At the RO, PI4KB III phosphorylates phosphatidylinositol (PI) to generate phosphatidylinositol-4-phosphate (PI4P) (15, 16). The balance between cholesterol and PI4P is critical because it maintains the specific membrane curvature required for proper assembly of the replication complex. In addition to cholesterol, sphingolipid metabolism is also hijacked by several RNA viruses, including CVB3. Sphingolipids contribute to the reduced fluidity and structural stability of ROs, providing a stable platform for anchoring the CVB3 polymerase (3D^pol^). Ceramide transfer proteins (CERTs), located at ER and Golgi membranes, function similarly to OSBP by transporting ceramides to the ROs, where they can be converted into sphingomyelin (SM) (17).

To broadly characterize metabolic changes in CVB3 infection, we performed global metabolomic analyses on CVB3-infected hepatocytes (Huh7) *in vitro*. We identified myriad lipid species in this screen, and we used this information to perform a targeted screen to investigate lipid metabolism inhibitors as potential antivirals. We identified the novel candidate AM095, an antagonist of lysophosphatidic acid receptor 1 (LPAR1). When we blocked LPAR1 signaling, either pharmacologically with AM095 or by gene knockdown, CVB3 titers decreased, and full-length viral genomes were no longer produced. Moreover, we found that inhibition of LPAR1 drives this replication defect by depleting host lipid metabolites within the fatty acid synthesis pathway. We found that AM095 activates AMPK signaling to reduce lipid synthesis through ACC1 and FASN. We further find that in cells treated with AM095 and replenished with the lipid palmitic acid, viral titers are fully rescued, suggesting that AM095’s antiviral activity is mediated by cellular lipid synthesis. Finally, we performed unbiased metabolomic analyses of cells infected with CVB3 *in vitro* and found that lipids are upregulated during infection. In sum, these findings highlight a new mechanism by which LPA signals through AMPK to mediate lipid synthesis, which highlights a novel lipid–virus interaction and suggests that targeting host lipid signaling through LPA signaling has antiviral potential.

## RESULTS

### Metabolic profiling of CVB3-infected cells

**Fig 1:**
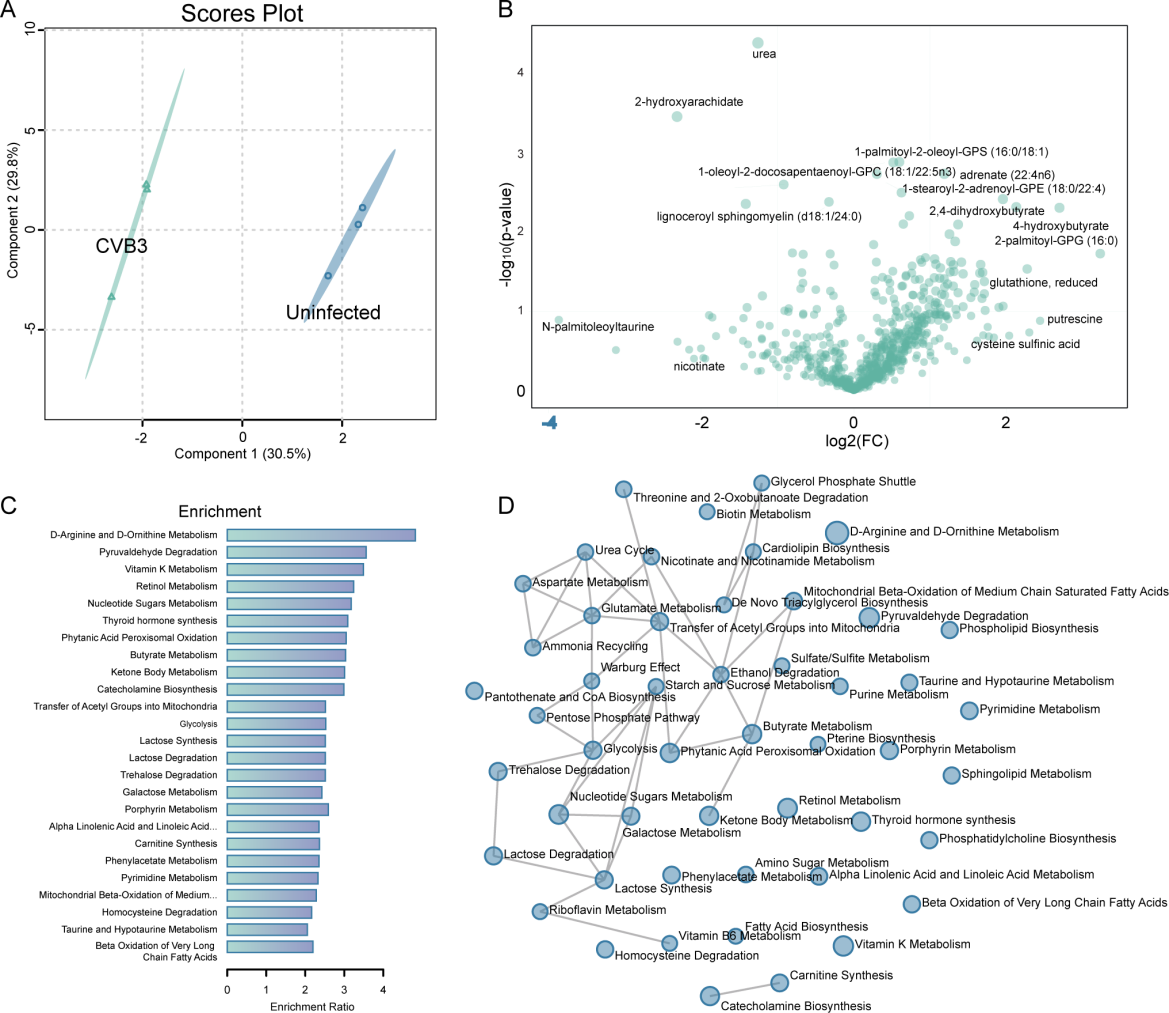
Metabolomic profiling of CVB3-infected Huh7 cells. Huh7 cells were seeded in 85% confluent flasks to generate ~10^7^ cells. Huh7 cells were infected at an MOI of 0.1 for 48 h. Following infection, cell pellets were spun down, frozen, and shipped to Metabolon Inc. Cell pellets were analyzed for metabolite amount and makeup using the Global (untargeted) Metabolomics HD4 panel. All analyses were performed on MetaboAnalyst. The following results compare uninfected and CVB3-infected Huh7 cells. (**A**) Score plot representing principal component analysis between uninfected and infected Huh7 cells. (**B**) Volcano plot of metabolites, dotted lines indicate fold change thresholds, and horizontal dotted lines represent statistical significance. The top 20 most significantly changed metabolites are noted. (**C**) Pathway enrichment analysis of the top 25 significantly enriched metabolic pathways. (**D**) Metabolic network analysis showing connectivity between pathways; lines represent shared metabolites between pathways. Error bars represent one standard error of the mean (*N* ≥ 3 for panels D–H). *P < 0.05, **P < 0.01, ***P < 0.001 by two-tailed Student’s *t*-test.

To globally characterize metabolite changes during CVB3 infection, we performed an unbiased metabolic screen using human Huh7 hepatocytes. Huh7 cells were specifically chosen due to their strong metabolic activity, specifically in lipid and energy metabolism (18). Cells were infected with CVB3 at a multiplicity of infection (MOI) of 0.1 for 48 h, harvested, and analyzed using the Global (untargeted) Metabolomics HD4 panel from Metabolon, generating a data set comprising 1,004 biochemicals. Metabolic data sets received from Metabolon were analyzed using MetaboAnalyst, an R-based online platform for statistical analysis, visualization, and pathway enrichment analysis (19–21). MetaboAnalyst analysis revealed striking shifts in metabolism between uninfected and CVB3-infected Huh7 cells, as expected. We first focused on global changes in metabolic profiles between sample types and generated a score plot to illustrate the largest variances between our two conditions (infected versus uninfected; Fig. 1A). We noted a clear separation between uninfected and CVB3-infected samples, with PC1 accounting for 30.5% and PC2 accounting for 29.8% of the total variance. Replicates of the same condition cluster near each other but do not overlap, reflecting slight variability. Overall, the score plot indicates a distinct metabolic reprogramming in Huh7 cells following CVB3 infection.

To identify the key metabolites driving the variance within the score plot, we performed differential analysis between uninfected and CVB3-infected Huh7 cells. The significantly altered metabolites were visualized using a volcano plot (Fig. 1B). Interestingly, over half of the most significantly altered metabolites are lipids. This finding aligns with the enterovirus literature, which shows that CVB3 and other enteroviruses rely on and manipulate lipids in multiple stages of their replication cycle (4, 8, 10, 18). The volcano plots highlight specific metabolite changes; however, to gain a comprehensive understanding of these alterations, we assessed metabolic pathway shifts via enrichment analysis. We performed pathway analysis to identify the top 25 pathways enriched during CVB3 infection in Huh7 cells (Fig. 1C). Consistent with the significantly altered lipid metabolites noted in the volcano plot, the pathway analysis highlighted enrichment of lipid-associated pathways, including beta-oxidation and energy metabolism.

We further explored how these metabolites within specific pathways interact by network analysis (Fig. 1D). In the network, connected lines represent shared metabolites between pathways. Several pathways form connected clusters within the network, including lipid-associated pathways, amino acid metabolism, energy metabolism, and sugar metabolism, reflecting the broad changes in metabolism observed upon pathway analysis. Together, these findings provide a comprehensive overview of the metabolic reprogramming that occurs during CVB3 infection in Huh7 cells and demonstrate the central role of lipids within the CVB3 infection process.

### Lipid changes in CVB3 infection

**Fig 2:**
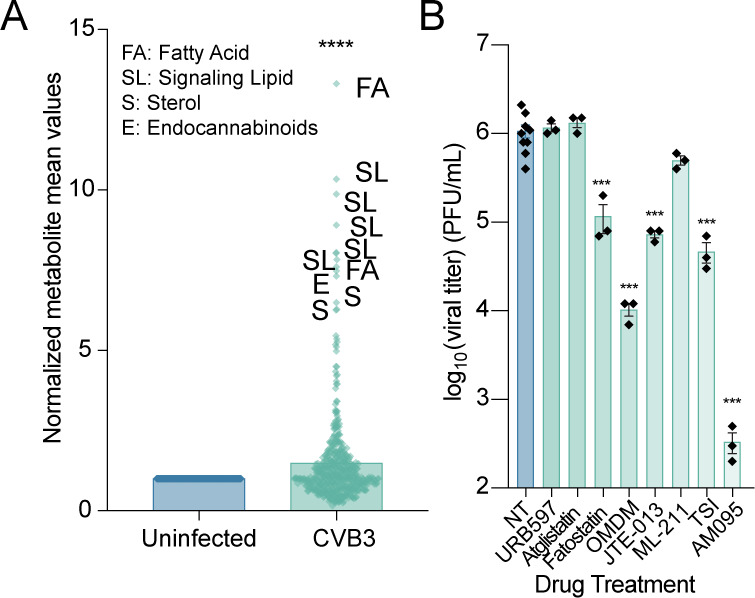
Lipid changes during CVB3 infection. (**A**) Mean metabolite concentrations representing only lipid metabolites, normalized uninfected Huh7 cells to 1. Top 10 hits are labeled by lipid class. (**B**) Drug screen was performed on Huh7 cells that were treated with compounds 24 h prior to infection, infected at an MOI of 0.01 of CVB3 for 48 h, and supernatant was collected for quantification via plaque assay. Lipid inhibitor treatments: URB597 (50 µM), atglistatin (20 µM), fatostatin (20 µM), OMDM (20 µM), JTE-013 (50 µM), ML-211 (20 µM), TSI (50 µM), and AM095 (100 µM). *P < 0.05, **P < 0.01, ***P < 0.001 by two-tailed Student’s *t*-test.

Lipids play key roles in viral replication, including for CVB3. Given the broad changes to lipid metabolism that we observed from our metabolomics screen, we chose to focus on this family of metabolites. To further investigate the changes in specific lipid metabolites during CVB3 infection, we compared the mean levels of lipid metabolites between uninfected and infected conditions. We observed that the mean concentrations of different lipid species are significantly higher in CVB3-infected Huh7 cells (Fig. 2A), supporting the volcano plot and enrichment pathway analysis results and highlighting significant changes in lipid metabolism during CVB3 infection. We considered the identities of individual lipids that were most significantly changed, which revealed several lipid groups that increased in abundance during CVB3 infection: sterols, endocannabinoids, fatty acids, and signaling lipids, highlighted on Fig. 2A. Importantly, although the abundance of these lipid groups increased during infection, these observations alone do not explain their functional importance.

Given the observed changes in these specific classes of lipid species, we performed a drug screen targeting either the biosynthesis of these lipids or their downstream functions, such as receptor-mediated signaling. We initiated a targeted drug screen with inhibitors of lipid synthesis and signaling including URB597 (fatty acid amid hydrolase inhibitor [22]), atglistatin (adipose triglyceride lipase inhibitor [23]), fatostatin (sterol regulatory element binding protein activation inhibitor [24]), OMDM-2 (anandamide uptake inhibitor [25]), JTE-013 (sphingosine-1-phosphate receptor 2 antagonists [26]), TSI-01 (lysophosphatidylcholine acyltransferase 2 inhibitor [27]), ML-211 (lysophospholipase 1 and 2 inhibitor [28]), and AM095 (LPAR1 antagonist [29]). To test the importance of these lipid-modulating drugs during infection, we treated Huh7 cells, infected with CVB3 at a multiplicity of infection (MOI) of 0.01 plaque-forming units (PFU) per cell, and performed a plaque assay to quantify viral titers following drug treatment after 48 h of infection (Fig. 2B). We observe sensitivity to several lipid inhibitors, including atglistatin, fatostatin, OMDM-2, JTE-013, ML-211, and AM095. AM095 treatment resulted in the greatest reduction in viral titer and was selected for further investigation.

### LPAR1 signaling inhibition limits CVB3 infection

**Fig 3:**
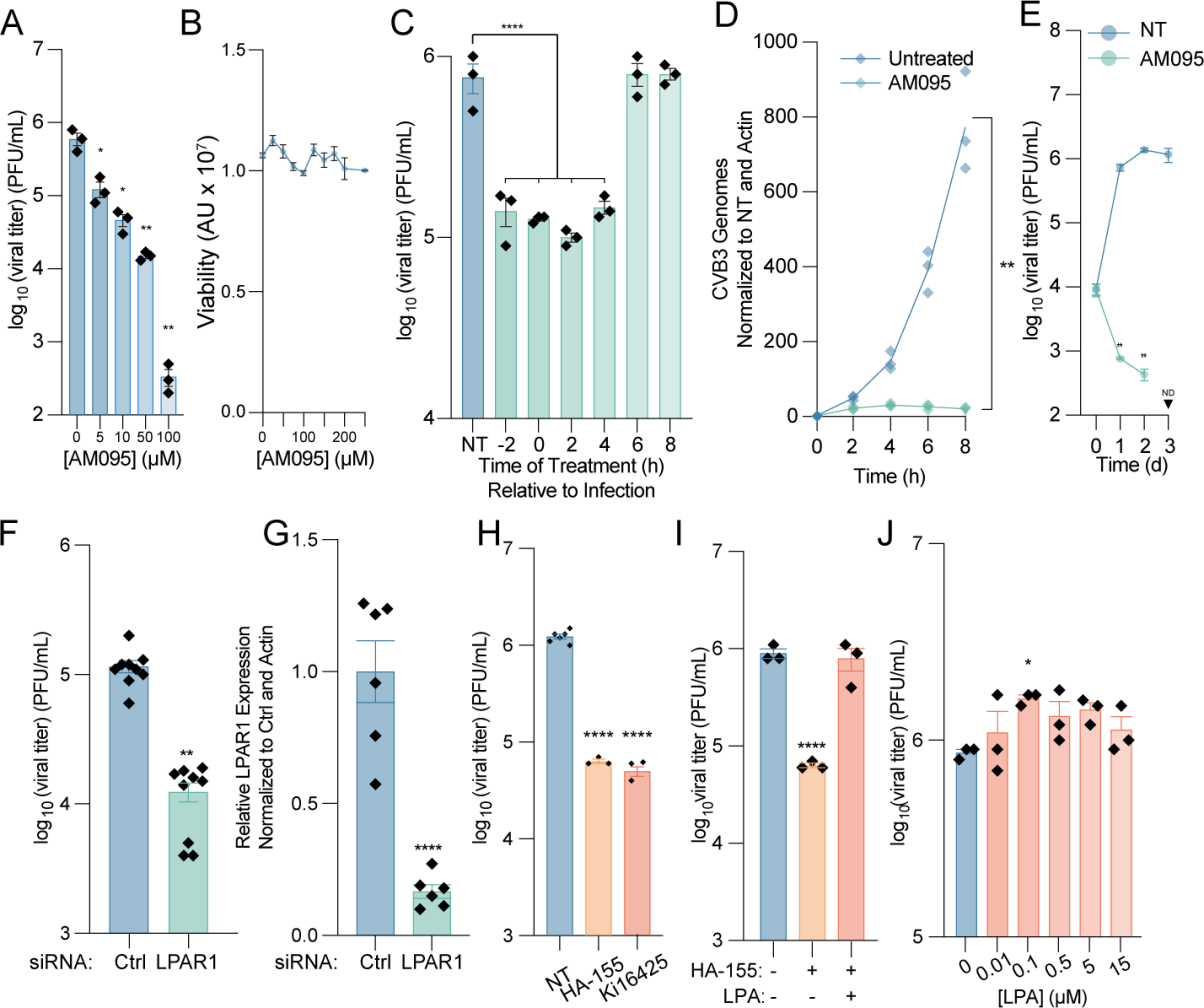
LPAR1 signaling is critical for CVB3 infection. All of the following experiments were performed on Huh7 cells that were treated with compounds 24 h prior to infection, infected at an MOI of 0.01 of CVB3 for 48 h, and supernatant was collected for quantification via plaque assay. (**A**) AM095 (5–100 µM). (**B**) Huh7 cells were treated with AM095 for 24 h, and cell viability was measured. (**C**) 100 µM AM095 treatment at indicated times (−2 h, –8 h) relative to infection time 0 h; (**D**) 100 µM AM095 treatment 24 h prior to cell collection, cDNA generation, and qPCR for CVB3 genome detection; (**E**) 100 µM AM095 treatment followed by daily collection up to 5 dpi. (**F**) Cells were transfected with specific to LPAR1 using oligofectamine and infected the following day for 24 h with CVB3 MOI 0.01. Supernatant was collected for plaque assay, and cells were collected for (**G**) qPCR verification; (**H**) 10 µM HA-155 and 100 µM Ki16425 treatment; (**I**) 10 µM HA-155 treatment 2 h prior to infection and supplemented with 0.5 µM LPA 1 h prior to infection. (**J**) Cells were supplemented with LPA 1 h prior to infection with increasing doses of 0.01 to 15 µM. Error bars represent one standard error of the mean (*N* ≥ 3 for panels D–G). *P < 0.05, **P < 0.01, ***P < 0.001 by two-tailed Student’s *t*-test

To follow up on the significant antiviral activity of the molecule AM095, we performed secondary analyses, focusing on the potent viral inhibitor AM095. We tested a range of doses from 5 to 100 µM and observed a dose-dependent reduction in CVB3 titers (Fig. 3A). Importantly, all experiments were performed in low (2%) serum, as LPA plays roles in the cell cycle, which affects CVB3 infection (30, 31). We used this low-serum media to control for potential effects of AM095 on the cell cycle (32). To ensure titer reduction was not due to cytotoxicity, we performed a viability assay on cells treated with increasing doses of AM095. Viability was determined via the CytoTox-Fluor cytotoxicity assay. Cells remain viable through treatment up to 250 μM (Fig. 3B), and, in fact, we were unable to calculate a CC50 due to the lack of observable cytotoxicity. To determine where in the viral life cycle AM095 was exerting its antiviral activity, we performed a time-of-addition (TOA) assay. We treated Huh7 cells at time −2, 0, 2 4, 6, and 8 relative to infection with 100 µM AM095. Following infection at MOI 10, viral titer was measured by plaque assay. We found that AM095 was most effective early in infection, up to 4 h post-infection, suggesting that AM095 acts at an early stage in infection (Fig. 3C). To expand on this, we analyzed the accumulation of viral genomes over the course of infection with 100 µM AM095 treatment. CVB3 genomes did not significantly accumulate in cells treated with AM095 as compared with untreated controls (Fig. 3D). Together, these results suggest that AM095 may be inhibiting early stage(s) in CVB3 infection.

We find that AM095 acts at early stages of infection and prevents viral genome accumulation. We next considered if the virus could overcome AM095 treatment after prolonged infection, with multiple rounds of replication. We performed an extended time course in which cells were treated with 100 µM AM095, infected with CVB3 at MOI 0.01, and collected over the course of three days for plaque assay analysis of infectious virus. In untreated cells, viral titers followed a classical sigmoidal curve, with new virus being produced at one dpi; however, cells treated with AM095 showed a significant reduction in viral titer after 24 h, with virus undetectable after 2 days post-infection (dpi) (Fig. 3E), suggesting that CVB3 is sensitive to sustained AM095 treatment.

To verify that the reduction in viral titer was due to LPAR1 inhibition and not off-target effects of AM095, we knocked down LPAR1 using siRNAs. siRNAs were transfected into 293T cells 24 h before CVB3 infection. Both supernatant and cell pellets were collected for viral quantification and knockdown verification. With LPAR1 knockdown, we observe a significant reduction in viral titer, corroborating our inhibitor-based data (Fig. 3F). We also verified LPAR1 knockdown by qPCR with primers specific to LPAR1 in comparison to our negative control condition, observing a significant reduction in LPAR1 mRNA levels (Fig. 3G), as expected.

We next examined if other drugs targeting the LPAR1 pathway could similarly inhibit CVB3. Huh7 cells were treated with HA-155 and Ki16425. HA-155 indirectly inhibits LPAR1 signaling by blocking autotaxin (ATX), the primary enzyme responsible for generating LPA (1, 33). By inhibiting ATX, HA-155 reduces LPA availability, limiting its concentration and preventing activation of LPAR1. In contrast, KI16425 is an antagonist for both LPAR1 and LPAR3 (34). Huh7 cells were treated with 10 µM HA-155 or 100 µM Ki16425, infected with CVB3, and viral titers were measured via plaque assay. Both treatments resulted in a significant reduction in viral titer (Fig. 3H). To confirm HA-155’s effect was due to the depletion of LPA availability, we supplemented LPA into the supernatant of HA-155 treated cells prior to infection, which fully rescued viral titers (Fig. 3I). Finally, to determine if LPA itself promotes CVB3 infection or if the downstream receptor-mediated signaling is responsible, we treated Huh7 cells with increasing doses of LPA, infected, and measured viral titer. LPA treatment alone had modest effects on CVB3 titers at any dose tested, though titers were significantly, yet modestly, enhanced at 0.1 µM LPA (Fig. 3J), suggesting that the LPAR1 signaling pathway, rather than LPA itself, is responsible for supporting CVB3 infection.

### AM095 inhibits CVB3 genome replication

**Fig 4:**
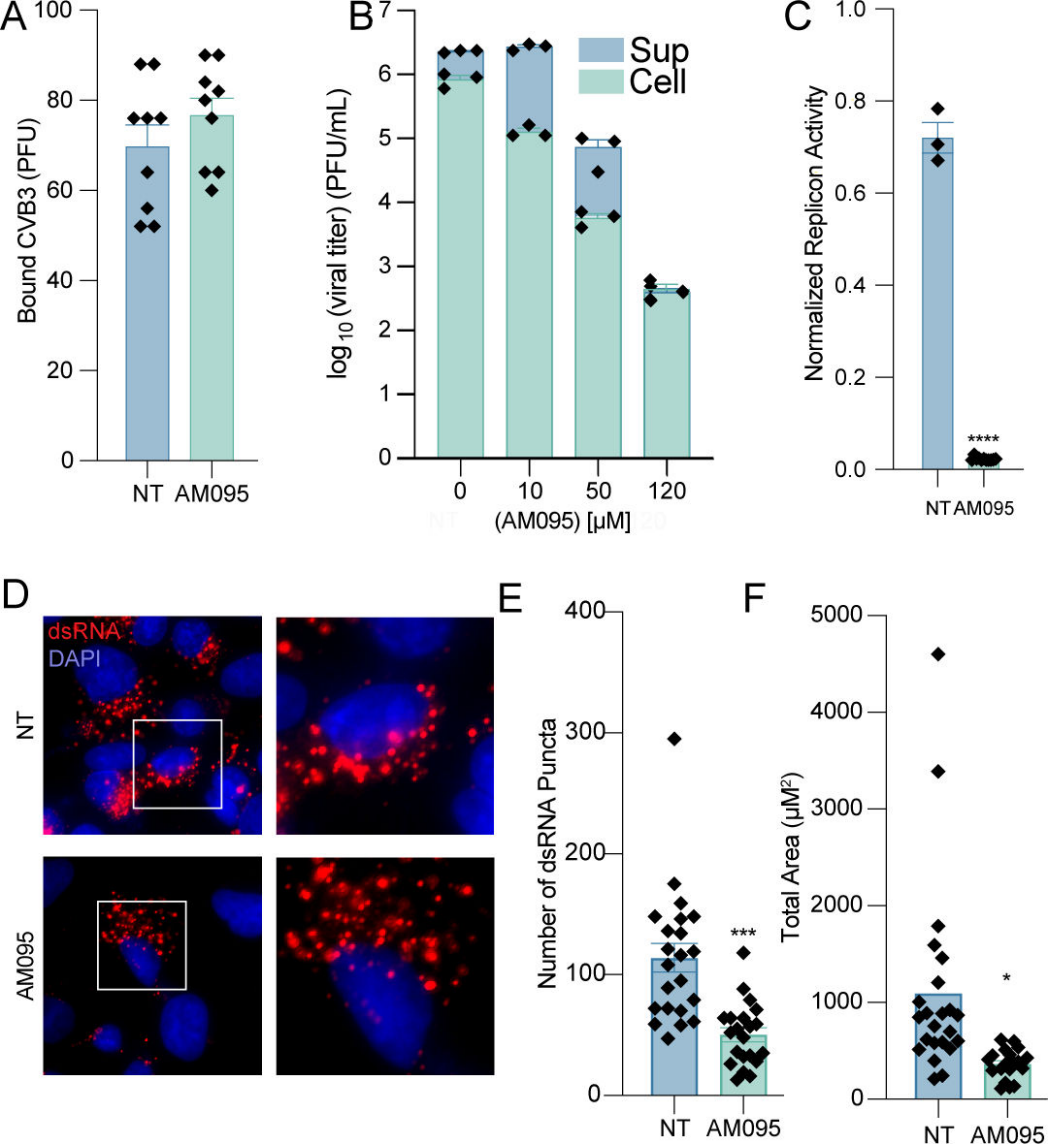
AM095 treatment inhibits CVB3 replication. (**A**) Vero E6 cells were treated with 100 µM AM095 2 h before infection with 1,000 PFU/m. Cells were placed on ice during infection for 5 minutes to prevent endocytosis. Virus was removed, and cells were washed with PBS, overlaid with 0.1% soft agarose, and incubated for 2 days at 37°C. Plates were fixed and stained using crystal violet. (**B**) Huh7 cells were treated with AM095 2 h before infection and infected at an MOI of 0.01 CVB3 for 48 h, and supernatant and cells were collected and quantified via plaque assay. (**C**) 293T cells were treated 2 h and transfected with CVB3 replicon. Luminescence was measured 24 h post-transfection and normalized to a transfection control. (**D**) Huh7 cells were infected for 6 h at MOI 10, then fixed and stained using antibody against double-stranded RNA. Cells were analyzed using a Ti2 Nikon Scope and analyzed using ImageJ software. (**E**) Quantified K1 punctuation from panel D via ImageJ. (**F**) Quantified total area of K1 puncta from panel D via ImageJ. Error bars represent one standard error of the mean (*N* ≥ 3). *P < 0.05, **P < 0.01, ***P < 0.001 by two-tailed Student’s *t*-test.

We next aimed to explore the mechanism by which AM095 inhibits CVB3 infection. Based on our time-of-addition data (Fig. 2C), AM095 appeared to act during early stages of the CVB3 life cycle. To test these events, we performed a binding assay to determine whether AM095 treatment affects CVB3’s ability to attach to cells. Cells were placed on ice and infected with 1,000 PFU of CVB3. The cells were maintained on ice for 5 min to allow viral binding without entry. After incubation, the unbound virus was washed away, and cells were overlaid with 0.1% soft agarose, without AM095 in the overlay. Following 2 days of incubation, the plates were stained, and plaques were quantified. Plaques observed under these conditions represent the amount of CVB3 that bound to the cell surface and successfully infected. We did not observe any changes in the number of plaques, suggesting that AM095 does not impact CVB3 attachment to cells (Fig. 4A). To ensure the reduction in viral titer was not due to impaired viral release, we performed an egress assay. Cells were treated with 100 μM AM095 and infected as described above. Virus was collected and quantified from both the supernatant, representing extracellular virus, and the cells, representing intracellular virus. We observed that AM095 decreased viral titers both internally and externally in a dose-dependent manner (Fig. 4B), suggesting that viral egress inhibition was an unlikely cause of reduced titers.

We next aimed to test whether AM095 impacts CVB3 replication. We performed a replicon assay on 293T cells treated with 100 μM AM095 and transfected with a luciferase-based CVB3-replicon plasmid, quantifying replication via luminescence analysis. Cells treated with AM095 exhibited a significant decrease in luminescence compared with untreated cells (Fig. 4C), indicating that AM095 inhibits CVB3 replicon-based luciferase activity. To further investigate this phenotype, we performed immunofluorescence (IF) assays to visualize viral replication complexes. Cells were treated with 100 µM AM095 and infected at MOI of 10 for 6 h. After infection, cells were fixed and stained with antibody to detect double-stranded RNA, a marker of active CVB3 replication, and stained with DAPI. We observed dsRNA-staining puncta in both untreated and AM095-treated conditions at six hpi (Fig. 4D). These puncta confirm that dsRNA is present, but at a reduced level consistent with previous data. Quantification of dsRNA staining across multiple images revealed a significant reduction in puncta number with AM095 treatment, and the puncta occupied less area, indicating that replication factories are smaller when LPAR1 signaling is blocked (Fig. 4E and F). These data collectively support that AM095 leads to reduced viral genome replication, leading to a decrease in virus titer. Importantly, this assay does not distinguish between specific steps in viral genome replication, such as protein translation or polymerase activity; however, our data suggest AM095 inhibits the overall process of genome replication.

### AM095 modulates lipid metabolism during CVB3 infection

**Fig 5:**
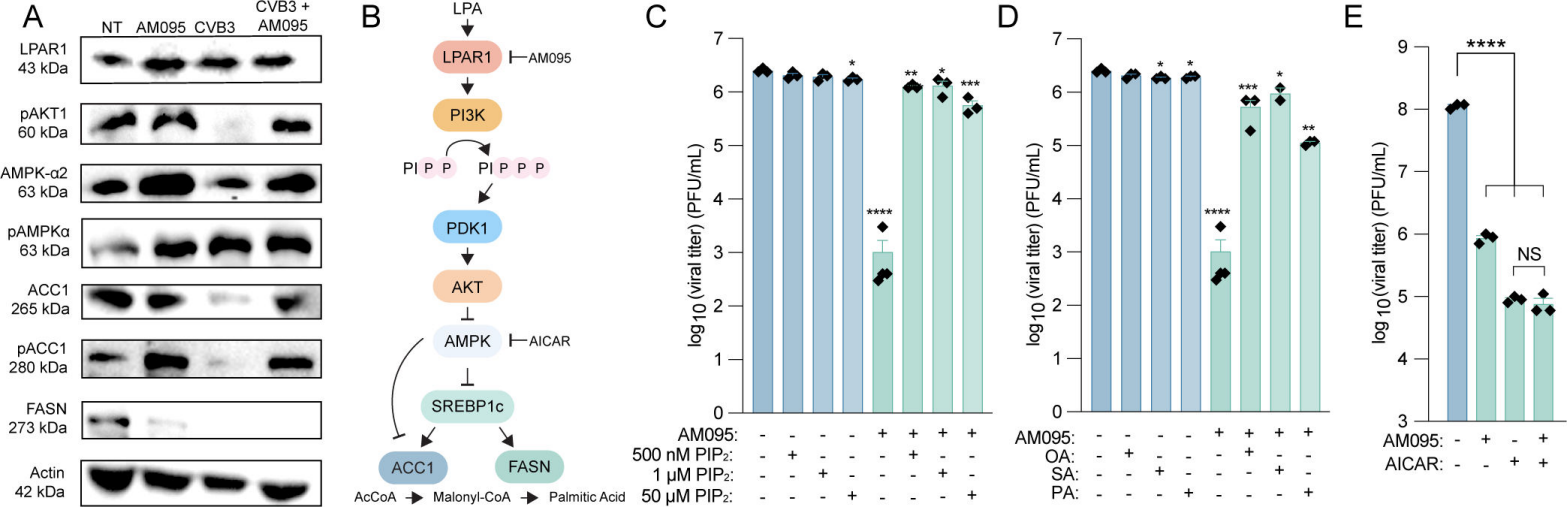
Lipid supplementation rescues the effects of LPAR1 signaling blockade. (**A**) Huh7 cells were treated with AM095 and infected with an MOI of 5 of CVB3 for 8 h. Cells were resuspended, and protein expression was measured via Western blot (**B**) Representation of inhibition by AM095 on LPAR1. LPAR1 antagonism will no longer inhibit AMPK activation, allowing it to activate. AMPK activation will phosphorylate enzymes like ACC1 or SREBP1c, leading to their inhibition. ACC1 can no longer convert Acetyl-CoA to Malonyl-CoA, leading to a reduction in fatty acid synthesis within the fatty acid biosynthesis pathway. Additionally, the regulation of fatty acid synthesis genes by SREBP1c will be inhibited. (**C and D**) Huh7 cells were treated with AM095 2 h before infection and supplemented with various lipids 1 h before infection. Lipids include 500 nM – 50 μM PIP_2_, 50 μM oleic acid (OA), 50 μM steric acid (SA), and 50 μM palmitic acid (PA). Cells were infected at an MOI of 0.01 of CVB3 for 48 h. Virus was quantified by plaque assay. (**E**) Huh7 cells were treated with 100 μM AM095 or 500 μM AICAR 2 h before CVB3 infection at an MOI of 0.01. At 48 hpi, virus was quantified via plaque assay. Error bars represent one standard error of the mean (*N* ≥ 3). *P < 0.05, **P < 0.01, ***P < 0.001 by two-tailed Student’s *t*-test.

We next considered the downstream signaling events that may mediate the observed phenotypes with CVB3 infection. To investigate this, we performed western blot analysis of AM095-treated and CVB3-infected cells. Cells were AM095-treated and infected as described for the immunofluorescence experiments. We first evaluated LPAR1 protein levels to determine whether AM095 alters receptor abundance. We observed a modest increase in LPAR1 under both treatment and infection conditions compared with NT (Fig. 5A), which may reflect a compensatory response to receptor blockade or stress induction by viral infection, consistent with reports of LPAR1 upregulation during other viral infections (2). Upon LPA binding to LPAR1, AKT1 serine/threonine kinase 1 (AKT1) becomes phosphorylated and active (summarized in Fig. 5B). We observed that AM095 treatment increased phospho-AKT1, but CVB3 infection abolished phospho-AKT1. This aligns with literature showing that host cells downregulate AKT1 signaling during viral infection as part of an apoptotic or stress response (35). AKT1 is known to phosphorylate monophosphate-activated protein kinase (AMPK) at its regulatory site at serine 487 (S487) (36), inducing a conformational change that prevents upstream kinases such as liver kinase B1 (LKB1) from phosphorylating the catalytic site, ultimately inhibiting AMPK activation (37). AMPK-α2 levels increased with AM095 treatment, potentially reflecting reduced AKT1-mediated inhibition. With CVB3 infection, AMPK-α2 decreased. Phosphorylated AMPK, however, increased across both infection and treatment conditions relative to untreated conditions, suggesting activation through stress responses that could contribute to viral inhibition by depleting cellular resources, or reflecting reduced CVB3 replication.

AMPK regulates lipid metabolism by phosphorylating and inactivating acetyl-CoA carboxylase 1 (ACC1), the rate-limiting enzyme for fatty acid synthesis (Fig. 5B) (38). We observed similar total ACC1 levels with AM095 treatment, but CVB3 infection reduced ACC1 expression. Notably, phosphorylated ACC1 increased with AM095 under both uninfected and infected conditions, supporting our proposed mechanism in which AM095 activates AMPK, leading to ACC1 inhibition and reduced lipid synthesis (Fig. 5B). Downstream of ACC1, we examined fatty acid synthase (FASN), which synthesizes lipid precursors critical for viral replication (39). FASN expression decreased with AM095 treatment and was abolished during CVB3 infection. This is consistent with inhibition of ACC1 by AMPK, which reduces malonyl-CoA availability and consequently inhibits FASN translation. Collectively, these findings support a model in which AM095 limits CVB3 replication by activating AMPK and inhibiting lipid metabolism.

To directly test whether signaling downstream of LPAR1 mediates AM095’s inhibition of CVB3 replication, we supplemented cells with phosphatidylinositol (4,5)-bisphosphate (PIP_2_), whose synthesis is regulated by LPAR1 signaling (Fig. 5B). Cells were treated with AM095 2 h before infection, supplemented with PIP_2_ 1 h before infection, and then infected with CVB3 at an MOI of 0.01 for 24 h. Viral titers were quantified using a plaque assay. We observed that PIP_2_ supplementation alone did not impact CVB3 viral titer (Fig. 5C), while treatment with AM095 caused a significant reduction in viral titer; interestingly, supplementation with PIP_2_ rescued viral replication, confirming that LPAR1 signaling specifically through PIP_2_ mediates at least part of the antiviral effect of AM095. Our Western blot data suggested that AM095 may alter fatty acid synthesis, which in turn may prevent CVB3 replication. We next supplemented cells with key fatty acids downstream of ACC1 and FASN (oleic acid, stearic acid, and palmitic acid; Fig. 5B). Alone, supplementation of each fatty acid only moderately (though significantly) reduced viral titers; however, in the presence of AM095, each fatty acid rescued viral replication (Fig. 5D), suggesting that these lipids mediate at least part of the antiviral effect of AM095. We observed subtle differences between the effectiveness of each of these lipids to rescue the phenotype, suggesting variability in either delivery, utility, or interconversion among these lipid molecules. Finally, to determine whether AMPK activation alone could recapitulate AM095’s antiviral effect, we treated cells with the AMPK activator AICAR, either alone or in combination with AM095. CVB3 titers were significantly reduced under all conditions, and AICAR and AM095 were not additive in their antiviral activity (Fig. 5E), supporting the model that phosphorylated AMPK is a key factor mediating AM095’s ability to limit viral replication.

### AM095 treatment reduces lipid content and reprograms cellular energy metabolism in CVB3-infected cells

**Fig 6:**
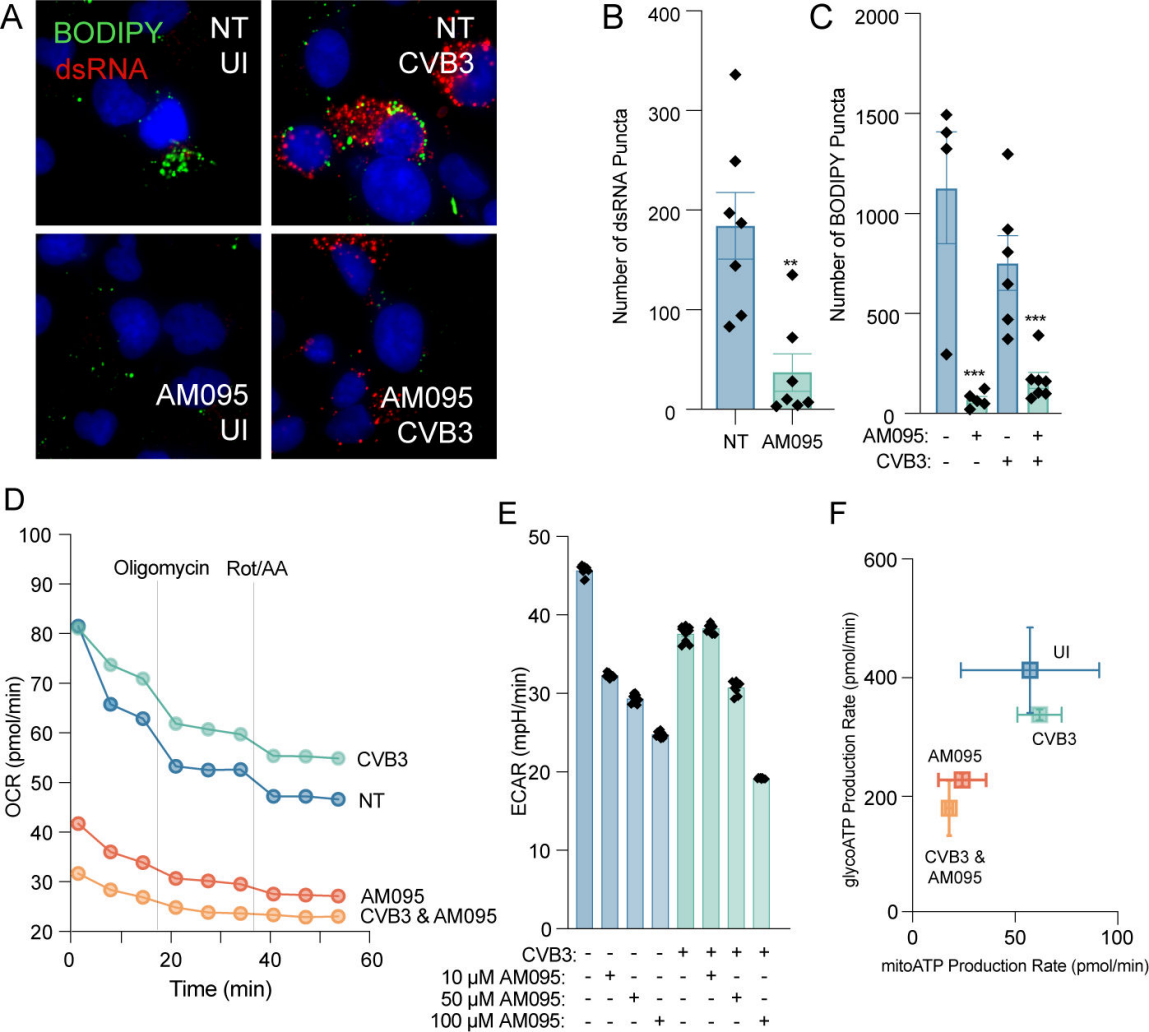
AM095 treatment reduces lipid content and reprograms cellular energy metabolism in CVB3-infected cells. (**A**) Huh7 cells were infected with CVB3 at MOI 10 for 6 h and then fixed and stained for lipid droplets (BODIPY) and viral dsRNA. (**B** and **C**) K1 and BODIPY puncta from IF images in panel A were quantified via ImageJ. (**D–F**) Huh7 cells were treated with AM095 2 h before infection with CVB3 at an MOI of 2 for 8 h. A Seahorse XF Real-Time ATP Rate Assay Kit was performed according to the Agilent user manual. The following OCR (**D**), ECAR (**E**), and energy map (**F**) were obtained from assay output. Error bars represent one standard error of the mean (*N* ≥ 3). *P < 0.05, **P < 0.01, ***P < 0.001 by two-tailed Student’s *t*-test.

To further investigate AM095’s impact on downstream lipid metabolism, we visualized lipid droplets by IF. We stained for double-stranded RNA and BODIPY, a lipid droplet stain. We visually observed a reduction in BODIPY staining in AM095-treated cells under both infected and uninfected conditions (Fig. 6A). We also observed a similar phenotype, with fewer infected cells, in our AM095-treated conditions. To verify these observations, we quantified the number of dsRNA and lipid droplet puncta and identified a significant reduction in dsRNA puncta in AM095-treated and infected samples compared with untreated controls, as well as a significant decrease in lipid droplet puncta in all AM095-treated cells (Fig. 6B and C). These findings support the hypothesis that AM095 treatment limits lipid metabolism to inhibit viral infection.

Since we observed a decrease in lipid production enzymes and lipid droplets, we next investigated the metabolic state of the cells. We used oxygen consumption rate (OCR) as a measure of metabolic activity via Seahorse assay on AM095-treated cells with CVB3 infection at MOI 5 for 8 h. Using the Agilent ATP Rate Assay kit, we injected two compounds: oligomycin, which inhibits ATP synthesis and decreases OCR, and rotenone/antimycin A (Rot/AA), which shuts down the electron transport chain and further reduces OCR. We confirmed proper assay function with working controls in untreated, uninfected cells (Fig. 6D). In uninfected samples, increasing doses of AM095 led to a dose-dependent reduction in OCR. We observed the same phenotype in CVB3-infected, AM095-treated cells. Interestingly, untreated infected samples exhibited a higher OCR at baseline and showed the greatest reduction with 100 µM AM095 compared with uninfected cells. These data indicate that AM095 alters cellular metabolic rates both in the presence and absence of infection. We found that CVB3 infection increased OCR; however, under conditions where AM095 inhibits LPAR1, CVB3 can no longer upregulate metabolic processes such as mitochondrial respiration.

We next assessed the extracellular acidification rate (ECAR) to complement the OCR results. ECAR reflects glycolytic activity, and we again observed that AM095 significantly reduces glycolysis. To visualize these results together, we generated an energy map to illustrate how cells balance their metabolic needs with AM095 treatment (Fig. 6F). The energy map plots OCR and ECAR, allowing discrimination of metabolic phenotypes: cells with low OCR and low ECAR are considered quiescent; cells with high OCR and low ECAR rely primarily on mitochondrial respiration; cells with low OCR and high ECAR rely primarily on glycolysis; and cells with high OCR and high ECAR are producing energy at high rates from both pathways. With this framework, we observe that cells treated with 100 µM AM095 fall within the quiescent quadrant. Lower doses of AM095 place cells in or near the quiescent quadrant compared with untreated, uninfected cells, which rely mainly on mitochondrial ATP. Untreated CVB3-infected cells appear in a mixed phenotype, utilizing both mitochondrial and glycolytic ATP generation. These data suggest that by altering lipid metabolism and reducing cellular energy production, AM095 effectively modulates the metabolic profile of both infected and uninfected cells, which we have shown to inhibit CVB3 replication through LPAR1 signaling.

## DISCUSSION

Enteroviruses, including Coxsackievirus B3 (CVB3), exploit host lipid metabolism to support multiple stages of their replication cycle. Previous studies demonstrated that CVB3 reprograms host metabolism to generate metabolites essential for infection, particularly those involved in lipid and energy pathways. Among these, the transcription factor SREBP1, which regulates genes involved in fatty acid synthesis, has been shown to play a critical role in viral replication (10). Our findings are consistent with these observations and provide additional evidence that CVB3 requires host-derived lipid metabolites as both an energy source and structural components for replication complex formation.

We observed that CVB3 infection significantly alters metabolic profiles in both Huh7 and Vero E6 cells, with pronounced changes in pathways related to lipid and energy metabolism. These alterations likely occur in a temporally regulated manner, reflecting the host cell’s adaptive response to infection and its attempt to restrict viral propagation. Inhibition of LPAR1 signaling, a pathway involved in lipid regulation, resulted in a significant reduction in viral titers. This phenotype was fully rescued upon supplementation with downstream fatty acids, directly supporting a lipid-dependent mechanism of CVB3 replication. Together, these data establish LPAR1 signaling as a critical host pathway for CVB3 infection and lipids as a downstream mediator of this phenotype.

Lysophosphatidic acid (LPA), the ligand for LPAR1, plays a central role in this process. LPAR1 signaling regulates diverse cellular functions, including membrane remodeling, immune modulation, and inflammation, all of which are known to influence viral infections. Additionally, LPA signaling mediates the cell cycle (32), potentially through the enhanced expression of cyclins (40). CVB3 infection is sensitive to the cell cycle (30, 31), and modulation of LPA signaling through AM095 could modify infection through the cell cycle. Our studies were specifically designed to use cells that were not cycling, using low-serum media, to control for effects of the cell cycle; however, future studies may specifically consider the potential mechanisms by which LPA signaling could contribute to viral infection through the cell cycle. Among currently available compounds, AM095 is the only selective LPAR1 antagonist. Consistent with our results, AM095 displayed no cytotoxicity and significantly reduced CVB3 infection *in vitro* for up to 5 days. These findings suggest that LPAR1 inhibition represents a viable therapeutic strategy for limiting CVB3 replication. In addition to LPAR1 inhibition, other aspects of this pathway require investigation. Autotaxin (ATX), the enzyme responsible for LPA synthesis, and other downstream effectors of LPAR1 signaling may represent additional targets for antiviral intervention. Interestingly, both ATX and LPA are currently under clinical investigation for fibrotic diseases, suggesting that existing pharmacological compounds could be repurposed for antiviral applications. This presents an exciting opportunity to evaluate these agents in the context of RNA virus infections, particularly in severe or persistent cases.

Mechanistically, viral infection is an energy-demanding process that requires substantial reprogramming of host metabolism (4). LPA is a key regulator of cellular energy balance, and LPAR1 signaling is known to activate the mTORC1 and MAPK pathways, which together promote lipid synthesis and anabolic metabolism (41–43). These pathways support the generation of membrane components crucial for the formation of the viral replication complex. CVB3 replication complexes rely on specific lipid species, including cholesterol, phosphatidylinositol 4-phosphate (PI4P), and various fatty acids (44, 45). Our findings suggest that LPAR1-associated lipid synthesis provides a source of these metabolites, supporting viral replication.

LPAR1 antagonism through AM095 treatment resulted in near-background measurements in our replicon assay. Viral titers could be rescued using distinct fatty acids, such as oleic acid, stearic acid, and palmitic acid, which are the initial fatty acids produced within the fatty acid synthesis pathway. Therefore, this suggests that fatty acid synthesis is critical for CVB3 replication in AM095-treated cells. Varied lipids can be derived from these initially-produced fatty acids, and many of them are critical components of lipid membranes, such as the membranes RNA viruses replicate on. These initial fatty acids can be converted into phospholipids (like phosphatidylinositol, phosphatidylcholine, or phosphatidylethanolamine), sphingolipids (like ceramides and sphingomyelin), and neutral lipids (like diacylglycerol). We hypothesize that, because replication is completely blocked and lipid supplementation rescues viral titer, the inhibition of LPAR1 and its downstream inhibition of lipid synthesis through AMPK phosphorylation of ACC1 prevents proper replication complex formation. In addition, this prediction is supported by our IF quantification, which shows that overall lipid droplets are decreased with AM095 treatment. Future studies will be needed to identify precisely how these replication complexes rely on specific lipid species, which is an active area of research.

Overall, our study identifies LPA and its receptor LPAR1 as proviral factors in CVB3 infection. This activity plays an integral role in regulating host lipid and energy metabolism to support viral replication. Beyond their metabolic functions, the identification of LPA as a novel facilitator of RNA virus replication highlights its potential as both a biomarker and a target for antiviral drug development. For example, circulating LPA levels may reflect the metabolic remodeling that accompanies viral infection (3). Future studies should focus on investigating the temporal dynamics of LPAR1 signaling during infection and defining the downstream metabolic pathways, including mTORC1 and MAPK, that contribute to viral replication for Coxsackieviruses and, more broadly, RNA viruses in general.

These insights could provide new therapeutic entry points for targeting RNA viruses that exploit host lipid metabolism. Importantly, targeting LPA and LPAR is under active clinical and preclinical consideration for the treatment of fibrosis, cancers, and other chronic diseases, suggesting that LPA-targeting therapies have significant potential *in vivo*. Further, several small-molecule LPA synthesis inhibitors and LPAR1 antagonists have demonstrated acceptable safety profiles in human trials (46–48), underscoring the clinical potential of these molecules. Repurposing these antagonists as antivirals represents an attractive host-directed therapeutic strategy, which may offer the benefit of reducing the evolution of antiviral resistance as compared with direct-acting antivirals, as the therapeutic target is a host factor rather than a viral target (49). In sum, our findings suggest that the LPA-LPAR1 axis is a compelling signaling node at the interface of host metabolism and RNA virus infection.

## MATERIALS AND METHODS

### Cell culture

Cells were incubated at 37°C, containing 5% CO_2_ in Dulbecco’s modified medium (DMEM; Life Technologies) containing bovine serum and penicillin-streptomycin. Vero E6 cells were supplemented with 10% newborn calf serum (NBCS; Thermo Fisher). Huh7 and HEK293T cells were supplemented with 10% fetal bovine serum (FBS; Thermo Fisher).

### Viability

Huh7 cells, unless otherwise noted, were seeded in a 96-well plate. Twenty-four hours later, cells were treated with increasing doses of AmM095. After 24 h of treatment, 50 μL of CytoTox-Fluor cytotoxicity assay reagent (Promega) was added to each well and incubated in the dark at room temperature (RT) for 45 min. Fluorescence of the plate was taken using a SpectraMaxiD3 fluorometer (485 nm excitation/520 nm emission).

### Metabolomics

Huh7 cells were seeded to 85% confluence in multiple T182 culture flasks. Cells were infected with CVB3 at an MOI of 0.1 for 24 h. Following infection, cells were removed from the flask using 0.25% trypsin and neutralized with 2% FBS or NBCS supplemented DMEM culture media. Samples were spun down for pellet collection, and supernatant was removed. Cell pellets were collected in tubes provided by Metabolon Inc. and shipped on dry ice overnight to Metabolon Inc. Whole cell metabolites were analyzed using the Global Discovery Panel. Visualization and interpretation of data were performed using web-based methods, including MetaboAnalyst and R software.

### Infection and enumeration of viral titers

CVB3 (Nancy strain) was derived from Vero E6 cells. Inhibitors were maintained throughout the course of infection as noted. For infection, virus was diluted in serum-free DMEM for an MOI of 0.01 on Huh7 cells unless noted otherwise in the figure legends. Viral inoculum was overlaid on cells for 30 min and washed with PBS before medium replenishment. Supernatant was collected at the times noted. Dilutions of cell supernatant were prepared in serum-free DMEM and used to inoculate a confluent monolayer of Vero E6 cells for 10 min at 37°C. Cells were overlaid with 0.1% agarose in DMEM containing 2% NBCS. Samples were incubated for 2 days, following appropriate incubation, cells were fixed with 4% formalin and stained with crystal violet solution (10% crystal violet; Sigma-Aldrich). Plaques were enumerated and used to back-calculate the number of plaque-forming units (PFU) per milliliter.

### Immunofluorescence imaging

Huh7 cells were seeded on coverslips and treated with 100 µM AM095 or untreated 2 h before CVB3 infection. Cells were infected with CVB3 MOI 5 for 8 h, then fixed with 4% formalin. Cells were blocked using a 0.2% Triton and 2% BSA in PBS at RT. Cells were washed using PBS and incubated with primary anti-dsRNA monoclonal antibody K1 (1:500; Kerafast) for 1 h at RT, followed by incubation with 5% BODIPY (Cayman Chemicals). Cells were washed with PBS and incubated with secondary goat anti-mouse antibody (1:1,000) for 1 h at RT. Slides were mounted onto coverslips using mounting media containing DAPI (Biotium) for nuclei visualization. As a control, uninfected samples were imaged as well. Samples were imaged with an ECLIPSE Ti2 Nikon microscope using a 60× objective. IF images were analyzed by ImageJ software.

### Western blot

Samples were collected using SDS buffer and run on 6 or 10% polyacrylamide gels. Gels were transferred in the Trans-Blot Turbo Transfer System (Bio-Rad). Membranes were blocked using 5% BSA in TBST and probed with primary LPAR1 (1:1,000; Invitrogen), Phospho-AKT1 (1:1,000; Abnova), AMPK-alpha2 (1:1,000; EMD Millipore Corp.), Phospho-AMPKalpha (1:1,000; Cell Signaling), Phospho-ACC1 (1:1,000; Cell Signaling), ACC1 (1:500; BioLegend), FASN (1:1,000; BioLegend), and actin (1:1,000; Protein Tech). Membranes were treated with Clarity Max Western ECL Substrate (Bio-Rad) and developed on a ChemiDoc imager (Bio-Rad).

### RNA extraction and qPCR

Cells were collected using Trizol reagent (Zymo Research). RNA was purified, DNase-treated (Zymo Research), and used for synthesizing cDNA with 5× All-In-One RT PCR Master Mix (BioBasic). cDNA was used for qPCR. A mix of CVB3-specific primers or control actin primers, SYBR (Zymo Research), and cDNA was used to plate a 96-well plate. CVB3 genome amounts were quantified via a real-time PCR machine (ThermoFisher). Primers used were (F) 3′-ATGCTTGCAAGGGTATGG-AATGGA-5′ and (R) 5' -ACGGTGAGGTGAACAGAA-TG-3′.

### Binding assay

Vero E6 cells were plated to confluence and treated with 100 μM AM095. Cells were plated on ice for 5 min and infected at a 1,000 PFU/mL with CVB3. Infected cells were kept on ice for 5 min, then washed 3× each with PBS to remove unbound virus. Cells were overlaid with 01% agarose and incubated at 37°C for 2 days. Cells were fixed using 4% formalin and stained with crystal violet.

### Egress assay

Huh7 cells were treated with 100 μM AM095 2 h before infection of CVB3 MOI 0.01 for 48 h. Cells and supernatant were collected for titer. For cell collection, the supernatant was collected, and the cells were washed with PBS 3×, and 500 μL of 1× PBS was placed on the cells. Cells were placed in a freezer for 5 min and removed 3× to ensure cell lysis. Both the supernatant and the cell samples were quantified for virus via plaque assay.

### Replicon analysis

Huh7 cells were treated with 100 μM AM095 2 h before transfection. Cells were transfected with T7 plasmid, CVB3 replicon plasmid (provided by Dr. Frank J.M. van Kuppeveld), and psiCheck transfection control (Promega). After 48 h, cells were lysed using passive lysis buffer, and Renilla or Firefly luciferase activity was analyzed using a microplate reader.

### Seahorse assay XF

Huh7 cells were seeded in a 96-well Seahorse XF Cell Culture Microplate and cultured in DMEM containing 2% FBS with 5% CO_2_ at 37°C. All drug treatments were performed as described above, 1 day after seeding cells. On the day of the assay, the cell culture growth medium was changed to Seahorse XF DMEM, supplemented with 1 mM pyruvate, 2 mM glutamine, and 10 mM glucose, and incubated in a 37°C non-CO_2_ incubator for 1 h before the assay. The sensor cartridge was loaded with 1.5 μM oligomycin and 0.5 μM rotenone + antimycin A. Samples were then run on the Agilent Seahorse XF Analyzer. Data were analyzed using Wave software.

### Statistical analysis

Prism 9 (Graphpad) was utilized to generate graphs and perform statistical analysis. For all analyses, ANOVA and two-tailed Student’s *t*-test were used to compare groups, unless otherwise noted with *α*  = 0.05.

### MetaboAnalyst 6.0 analysis

Metabolon Inc. files were uploaded into MetaboAnalyst through statistical analysis (one factor), statistical analysis (metadata table), statistical meta-analysis, enrichment analysis, pathway analysis, network analysis, and causal analysis. Metabolon XLXS files were uploaded, and prompts were followed for R-code generation. Graphs were exported into Adobe Illustrator or PDF files.

## Data Availability

Metabolomics data have been deposited in Zenodo with the DOI 10.5281/zenodo.17418525.
